# Short-term effects of oral dissolving film on halitosis and oral pathogenic bacteria: a pilot non-randomized controlled trial

**DOI:** 10.3389/fmicb.2025.1639960

**Published:** 2025-08-19

**Authors:** Mu-Yeol Cho, Je-Hyun Eom, Eun-Mi Choi, Ji-Won Kim, Young Youn Kim, Seung-Jo Yang, Ju-Young Hwang, Hye-Sung Kim

**Affiliations:** ^1^Apple Tree Institute of Biomedical Science, Apple Tree Medical Foundation, Goyang-si, Republic of Korea; ^2^DOCSMEDI, Co., Ltd., Goyang-si, Republic of Korea

**Keywords:** halitosis, oral dissolving film, tongue cleaner, volatile sulfur compounds, oral bacteria

## Abstract

**Introduction:**

Halitosis is a major issue that negatively affects individuals' social interactions and quality of life. This study aimed to evaluate the effects of oral dissolving film (ODF) on halitosis and oral pathogenic bacteria.

**Objective:**

To compare and evaluate the effects of ODF and tongue cleaner on reducing halitosis-related compound concentrations and oral pathogenic bacteria.

**Methods:**

A pilot, single-center, non-randomized, before-after comparative study was conducted with 30 adults with halitosis. The experimental group (n=15) consumed ODF three times daily for 7 days, while the control group (*n* = 15) used a tongue cleaner for the same period. H_2_, H_2_S, CH_3_SH, and total VSCs were measured using a halitosis analyzer, and seven oral pathogenic bacterial species were analyzed by qPCR.

**Results:**

Due to poor compliance, one participant from each group dropped out, resulting in 28 participants included in the final analysis. In the ODF group, H_2_ and CH_3_SH significantly decreased by 55.1% and 54.4%, respectively (*p* < 0.05), and *Streptococcus mutans* also significantly decreased by 27.9% (*p* = 0.022). The control group did not reach statistical significance for any halitosis parameters, and *Treponema forsythia* significantly increased by 5.8% (*p* = 0.047).

**Conclusion:**

ODF exhibited significant reductions in key halitosis-associated compounds and demonstrated antimicrobial activity against cariogenic bacteria. This suggests ODF may serve as a convenient alternative approach for both halitosis management and dental caries prevention. Further large-scale, randomized trials are warranted to confirm these preliminary findings. This study was registered in https://trialsearch.who.int (KCT0010244).

## Introduction

Halitosis represents a significant clinical condition that adversely impacts individuals' social interactions and quality of life (Setia et al., 2014; Olszewska-Czyz et al., 2022). The etiology of halitosis encompasses multiple factors, including insufficient oral hygiene, periodontal disease, tongue coating, and specific dietary habits (Kapoor et al., 2016). In particular, volatile sulfur compounds (VSCs) produced by anaerobic bacteria in the oral cavity are known as the main causative agents of halitosis. VSCs include hydrogen sulfide (H_2_S), methyl mercaptan (CH3SH), and dimethyl sulfide [(CH3)_2_S] (Tangerman and Winkel, 2010). Therefore, VSC reduction through oral bacterial management holds significant implications not only for halitosis reduction but also for oral health, quality of life, and social interactions.

Various methods have been proposed for halitosis management, including mechanical plaque removal, chemical antimicrobial agents, and masking agents (Kapoor et al., 2016). Nevertheless, these conventional approaches demonstrate inherent limitations in terms of duration of effect or side effects, highlighting more effective and safer alternative approaches. Recent research has suggested that innovative delivery systems such as oral dissolving films (ODF) may be effective in alleviating halitosis (Susanti et al., 2024). These formulations offer several clinical advantages, including rapid dissolution in the oral cavity for effective delivery of active ingredients, ease of use, and portability (Nagaraju et al., 2016).

Contemporary oral healthcare research has increasingly focused on naturally derived ingredients, which can contribute to minimizing side effects of synthetic chemicals and improving oral health. Among these natural ingredients, *Spilanthes acmella*, commonly known as the toothache plant, is a medicinal herb with established antimicrobial properties. The extract of this plant is known to have anti-inflammatory, antimicrobial, and analgesic effects, making it useful for promoting oral health (Prachayasittikul et al., 2013). Additionally, probiotics play an important role in maintaining oral health by regulating the microbial balance in the oral cavity and inhibiting the growth of harmful bacteria (Cho et al., 2025). A previous investigation developed an ODF by incorporating nanoparticulated mangosteen extract into a pullulan-alginate film, which demonstrated antimicrobial activity against *Streptococcus mutans, Streptococcus sanguinis*, and *Porphyromonas gingivalis* (Jittinan et al., 2013). However, research on the effects of ODF containing both *S. acmella* extract and probiotics on halitosis and oral pathogenic bacteria inhibition remains limited.

Therefore, this pilot study aimed to evaluate the short-term effects of ODF containing *S. acmella* extract and probiotics on halitosis and oral pathogenic bacteria. Through this preliminary investigation, we aimed to determine whether the film could serve as an alternative therapeutic approach for halitosis management and to establish foundational data for future larger-scale clinical trials.

## Materials and methods

### Study design and participants

**Figure 1:**
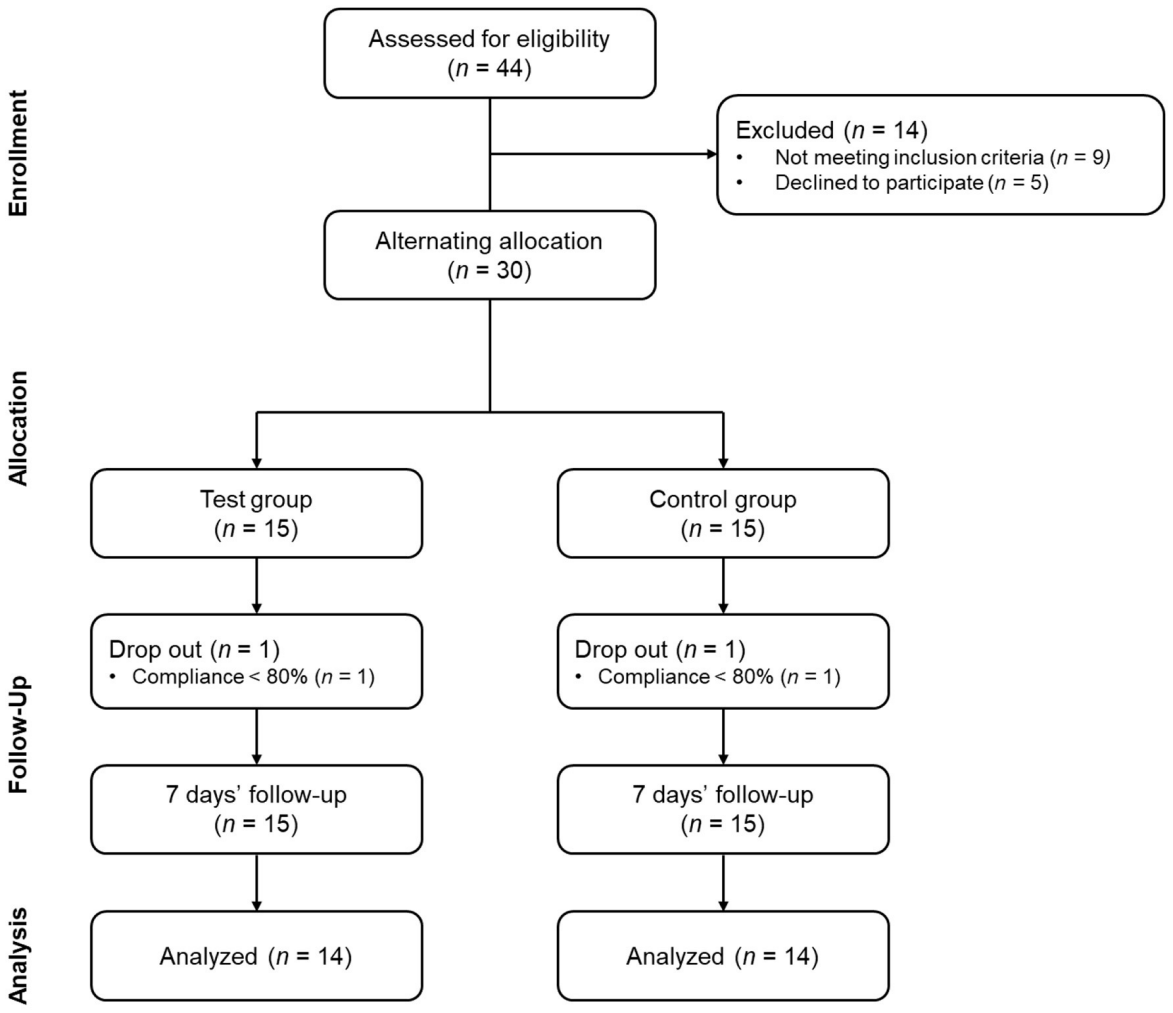
Flow chart of the study design.

This study was a pilot, single-center, non-randomized, before-after comparative study (Figure 1). The study was approved by the Institutional Review Board of Apple Tree Medical Foundation (approval number: ATDH-2024-0009) and registered in https://trialsearch.who.int (KCT0010244). Study participants comprised adults aged 18–70 years presenting with halitosis. Eligible individuals demonstrated H_2_S concentrations between 1.5 and 12 ng/10 mL or CH3SH concentrations between 0.5 and 9 ng/10 mL as measured by the halitosis assessment device (Kim and Kang, 2023). The threshold limits of 12 for H_2_S and 9 for CH3SH were established to prevent extreme outliers identified in our preliminary research and to reduce baseline differences between groups, as suggested by a previous study (Han et al., 2023). Inclusion criteria comprised three essential requirements. First, participants had no history of halitosis-related product usage within 7 days prior to enrollment. Second, voluntary informed consent for study participation was required. Third, participants must have demonstrated the capacity to maintain consistent lifestyle habits throughout the study duration. Power calculations were conducted to determine the appropriate sample size required to detect treatment effectiveness in a full-scale clinical trial. The statistical calculations demonstrated that a minimum sample size of 79 participants was required (approximately 40 per treatment arm). This sample size would provide adequate power to detect an absolute effect size difference of 5 units in VSC levels between treatment groups. The analysis assumed 80% statistical power and a significance level of α = 0.05. The rationale for conducting this pilot investigation with a limited sample size encompassed two primary considerations. First, we sought to determine whether a comprehensive full-scale clinical trial would be warranted based on observed trends and estimated effect sizes from our preliminary analysis. Second, given the paucity of published research on oral dissolving films for halitosis management, establishing preliminary evidence for this novel therapeutic modality was considered a valuable contribution to the scientific literature.

Exclusion criteria comprised several categories of participants. First, current smokers and individuals who had used oral dissolving films within 7 days were excluded. Second, participants requiring treatment for severe dental conditions (periodontitis, dental caries, xerostomia, peri-implantitis) were ineligible. Third, individuals currently receiving treatment for severe systemic diseases were excluded, including cardiovascular, immune, respiratory, gastrointestinal/biliary, neurological, musculoskeletal, infectious diseases, or malignant tumors. Fourth, pregnant women, women preparing for pregnancy, lactating mothers, and women within 6 months postpartum were excluded. Finally, individuals with known allergies to *S. acmella* extract or probiotics were deemed ineligible. All consent procedures and data collection were conducted in a designated independent space within the hospital. This standardized location was maintained throughout the entire study duration. Eligible participants were alternately assigned to treatment groups based on recruitment order. The experimental group received ODF treatment, while the control group used tongue cleaners. This alternating allocation method was selected because the interventions differed substantially in their application methods. Consequently, blinding was not feasible for either participants or researchers.

### Intervention

The experimental group was provided with ODF (Docsbiome OraFill, DOCSMEDI Co., Ltd., Republic of Korea). The composition of the ODF included *S. acmella* extract powder (*S. acmella* leaf extract, dextrin), pullulan, pectin, xylitol, flavoring (natural lemon flavor), *Lacticaseibacillus rhamnosus* DM163, glycerin, modified starch, steviol glycosides, citric acid, glycerin fatty acid ester, D-sorbitol, polysorbate 80, green tea extract, DL-malic acid, sage leaf extract powder, banaba leaf extract powder, pear concentrate, L-menthol, vitamin C, and sucralose. The control group was provided with a tongue cleaner product (WD700, Widen Tongue Cleaner, Widen Co., Ltd., Republic of Korea). The experimental group consumed one ODF (172.9 mg) three times daily (after breakfast and brushing, after lunch and brushing, and before bedtime after brushing) for 7–10 days by placing it on the tongue and allowing it to dissolve slowly. Oral hygiene management was not performed immediately after consumption. The control group used the tongue cleaner for 15 seconds three times daily (after breakfast and brushing, after lunch and brushing, and before bedtime after brushing) for the same period. The tongue cleaner was instructed to be used with moderate and tolerable pressure, scraping from the inside of the tongue toward the outside of the oral cavity (Casemiro et al., 2008). Both intervention products were delivered by a single researcher in the laboratory, and incentives in the form of transportation fees were provided to enhance compliance.

### Outcome measurements

Study participants were evaluated before product use and after 7–10 days of use. All visits were conducted between 07:00–13:00, and participants were instructed to maintain fasting for at least 8 h, avoid brushing teeth, and avoid oral fluid contamination on the day of the visit.

Primary outcome variables measured were total VSCs (H_2_S + CH3SH), H_2_, H_2_S, and CH3SH. Measurements were performed using a halitosis analyzer (Twin Breasor II™, iSenLab Inc., South Korea), and the average of two consecutive measurements was used for analysis. Secondary outcome variables included DNA copy numbers of pathogenic oral bacteria, which were analyzed by extracting genomic DNA from mouthwash samples using the Bacteria Genomic DNA Isolation Kit (LaboPass™, Cosmogenetech, Korea), followed by quantitative polymerase chain reaction (qPCR) analysis of seven bacterial species (*P. gingivalis, Tannerella forsythia, Treponema denticola, Prevotella intermedia, Campylobacter rectus, Fusobacterium nucleatum*, and *S. mutans*) as previously reported (Hwang et al., 2024).

Lifestyle habits and oral hygiene practices were assessed using a self-administered questionnaire adapted from the Korea National Health and Nutrition Examination Survey, a standardized and validated national survey conducted by the Korean Ministry of Health and Welfare (Kim and Chung, 2025). The questionnaire included binary (yes/no) questions regarding oral hygiene practices (interdental brushing, flossing, gargling, oral irrigator use, tongue cleaner use) and general lifestyle habits (alcohol consumption, exercise), as well as daily brushing frequency. All questionnaires were administered in a standardized manner within the same clinical environment, with research personnel providing explanations of each item prior to completion to ensure participant comprehension.

### Statistical analysis

Statistical analysis was performed only on data from participants who completed the study according to the protocol without protocol violations or dropouts. For primary and secondary outcome variables, normality testing was conducted to determine whether parametric or non-parametric tests should be used. Paired *t*-test was performed if data followed a normal distribution, and the non-parametric Wilcoxon Signed-Rank Test was used when the data did not follow normal distribution. Differences in age, daily brushing frequency, and compliance between groups were compared using Student's *t*-test. Fisher's exact test was performed for gender and the use of other oral care products. The significance level was set at 0.05. All analyses were performed using R Studio version 4.4.1 software.

## Results

### Participant characteristics

The recruitment and follow-up period lasted approximately 5 months from September 4, 2024, to February 13, 2025. A total of 28 participants (ODF 14, Tongue cleaner 14) completed the study participation and were analyzed. During the study period, there was one dropout in each group. The reason for dropout was poor compliance in all cases. No adverse events were reported. There were no statistically significant differences in demographic characteristics and lifestyle habits between the two groups (Table 1).

**Table 1:** Baseline characteristics and oral hygiene habits of the subject in the oral dissolving film and tongue cleaner groups.

**Variables**	**ODF (*n* = 14)**	**Tongue cleaner (*n* = 14)**	***p*-value**
Age (years)	52.8 ± 10.5	52.5 ± 7.9	0.936
**Sex**			0.222
Male	3 (21.4)	0 (0.0)	
Female	11 (78.6)	14 (100.0)	
**Interdental brushing**			1.000
Yes	5 (35.7)	4 (28.6)	
No	9 (64.3)	10 (71.4)	
**Flossing**			1.000
Yes	3 (21.4)	3 (21.4)	
No	11 (78.6)	11 (78.6)	
**Gargling**			1.000
Yes	1 (7.1)	1 (7.1)	
No	13 (92.9)	13 (92.9)	
**Oral irrigator**			1.000
Yes	0 (0.0)	1 (7.1)	
No	14 (100.0)	13 (92.9)	
**Tongue cleaner**			1.000
Yes	2 (14.3)	2 (14.3)	
No	12 (85.7)	12 (85.7)	
**Alcohol consumption**			0.596
Yes	3 (21.4)	1 (7.1)	
No	11 (78.6)	13 (92.9)	
**Exercise**			1.000
Yes	6 (42.9)	6 (42.9)	
No	8 (57.1)	8 (57.1)	
Brushing frequency (times/day)	2.2 ± 0.4	2.4 ± 0.5	0.241
Compliance (%)	96.1 ± 5.1	95.8 ± 9.2	0.899
Intervention duration (days)	8.5 ± 1.1	8.5 ± 0.9	1.000

Data are presented as mean ± standard deviation for continuous variables and number (percentage) for categorical variables. *P*-values were calculated using independent *t*-test for continuous variables and Fisher's exact test for categorical variables.

### Halitosis-related compounds

**Figure 2:**
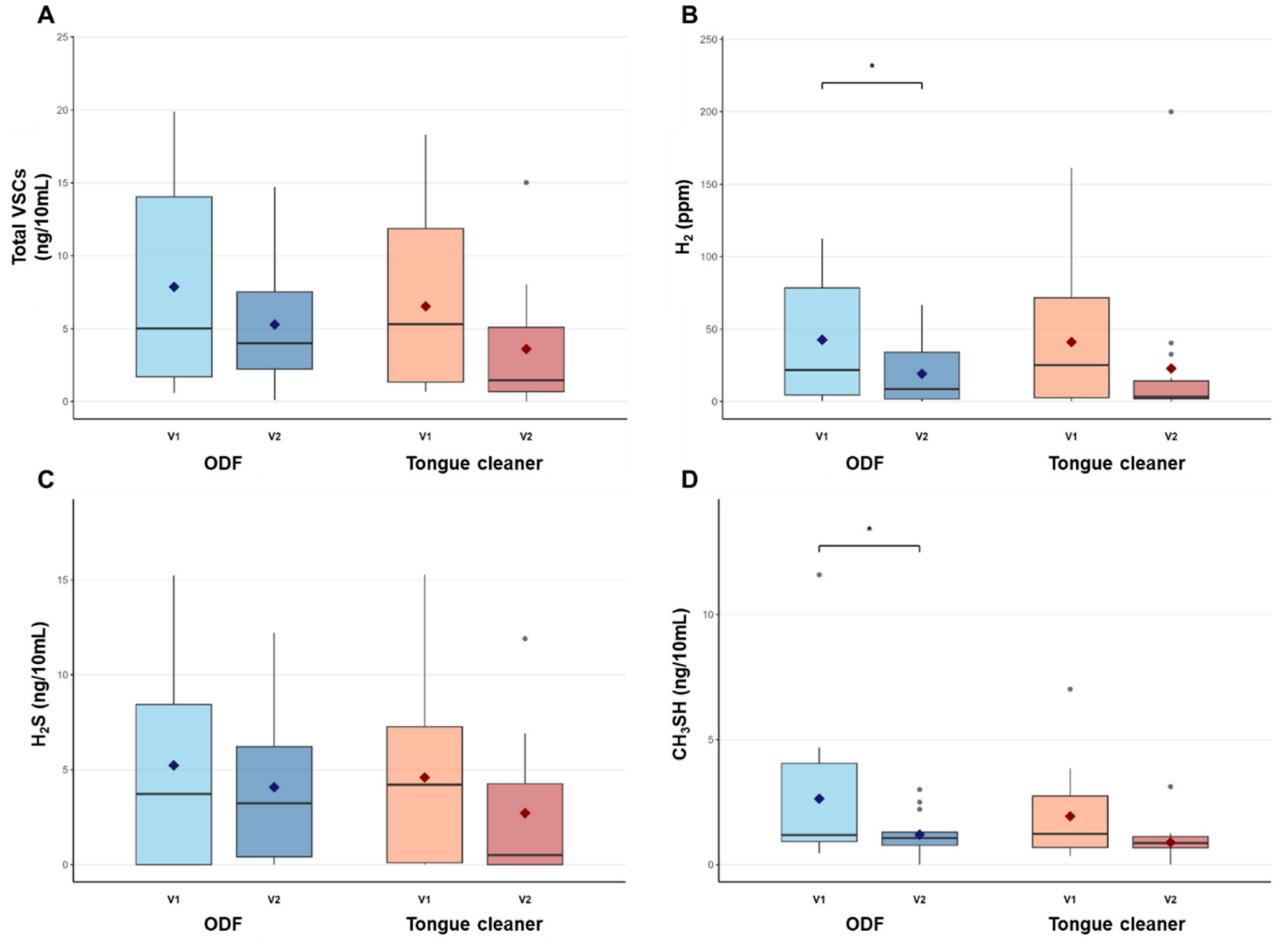
Pre- and post-intervention levels of halitosis-related compounds in ODF and tongue cleaner groups. Box plots show median (horizontal line), interquartile range (box), and mean (diamond) for total VSCs **(A)**, H_2_ **(B)**, H_2_S **(C)**, and CH_3_SH **(D)** in ODF (blue) and tongue cleaner (orange) groups. *p < 0.05 indicates significant within-group change. *n* = 14 per group.

Both treatment groups showed reductions in VSCs levels after intervention (Figure 2). At baseline, no significant differences were observed between the ODF and tongue cleaner groups for any measured parameter (total VSCs: *p* = 0.596, H_2_: *p* = 0.929, H_2_S: *p* = 0.748, CH_3_SH: *p* = 0.469), indicating comparable starting conditions between groups.

In the ODF group, total VSCs and H_2_S showed reduction of 32.84% and 21.96%, respectively, but these changes were not statistically significant (*p* = 0.131 and *p* = 0.396). H_2_ decreased significantly from 42.6 ± 43.7 ppm to 19.2 ± 21.9 ppm (55.06% reduction, *p* = 0.044), and CH_3_SH reduced from 2.6 ± 3.0 ng/10 mL to 1.2 ± 0.8 ng/10 mL (54.45% reduction, *p* = 0.045).

The tongue cleaner group demonstrated increase across all parameters, with reductions ranging from 40.91% to 54.01%, but none of these changes reached statistical significance (total VSCs: *p* = 0.144, H_2_: *p* = 0.264, H_2_S: *p* = 0.219, CH_3_SH: *p* = 0.079). When comparing treatment effects between groups, no significant differences were observed in the magnitude of change for any measured parameter (all *p* > 0.05). Similarly, post-treatment comparisons between groups showed no significant differences (total VSCs: *p* = 0.318, H_2_: *p* = 0.812, H_2_S: *p* = 0.352, CH_3_SH: *p* = 0.322).

### Oral microbial profiles

**Figure 3:**
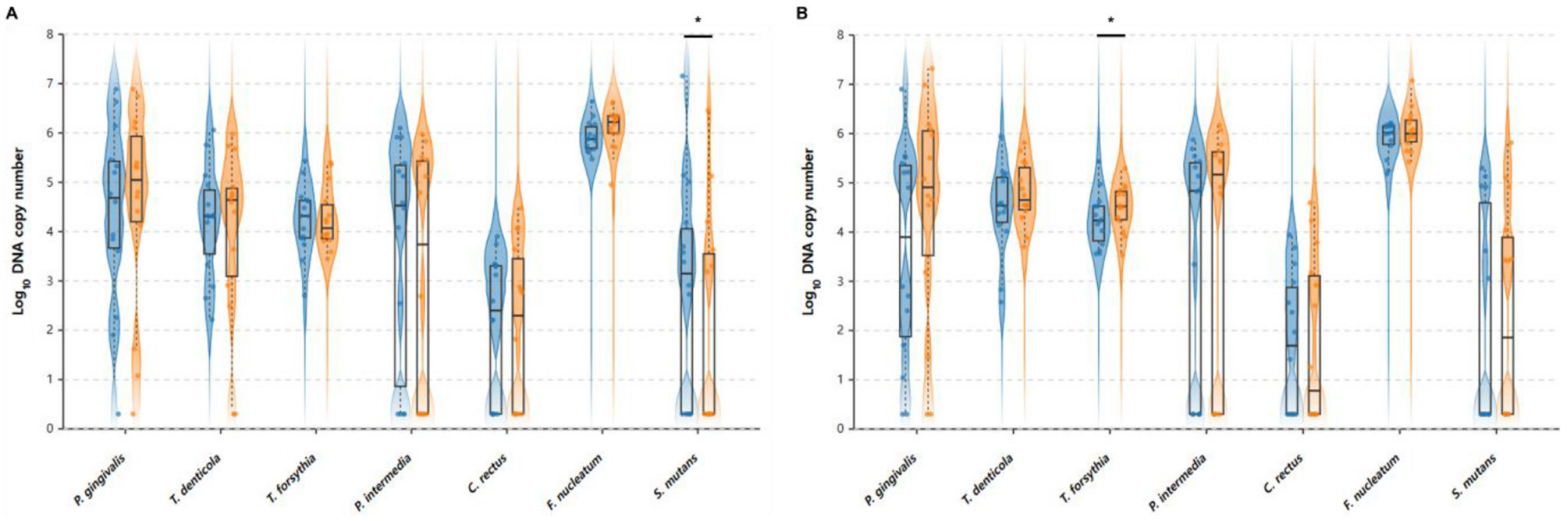
Pre- and post-intervention oral bacterial profiles in **(A)** ODF and **(B)** tongue cleaner groups. Violin plots showing the distribution of oral bacterial levels before and after intervention. Left (blue) violins represent baseline levels and right (orange) violins represent post-intervention levels. Box plots within each violin show the median (centerline), interquartile range (box), and whiskers extending to 1.5 times the interquartile range. Individual data points are shown as dots. *Indicate statistically significant differences between V1 and V2 within each group (*p* < 0.05, paired *t*-test).

The ODF group showed minimal changes in most bacterial species (Figure 3). *P. gingivalis* levels remained stable from 4.346 to 4.503 log_10_ DNA copy number (+3.6%, *p* = 0.572). *T. denticola* decreased from 4.212 to 3.939 log_10_ DNA copy number (−6.5%, *p* = 0.303), and *T. forsythia* remained nearly unchanged from 4.241 to 4.264 log_10_ DNA copy number (+0.5%, *p* = 0.850). *P. intermedia* decreased from 3.610 to 3.038 log_10_ DNA copy number (−15.8%, *p* = 0.269), *C. rectus* remained stable from 2.087 to 2.024 log_10_ DNA copy number (−3.0%, *p* = 0.815), and *F. nucleatum* increased slightly from 5.927 to 6.106 log_10_ DNA copy number (+3.0%, *p* = 0.118). *S. mutans* showed a significant reduction from 2.806 to 2.022 log_10_ DNA copy number (−27.9%, *p* = 0.022).

In contrast, the tongue cleaner group demonstrated increases in most bacterial species. *P. gingivalis* increased from 3.573 to 4.450 log_10_ DNA copy number (+24.5%, *p* = 0.135), *T. denticola* increased from 4.466 to 4.779 log_10_ DNA copy number (+7.0%, *p* = 0.135), and *T. forsythia* showed a significant increase from 4.256 to 4.501 log_10_ DNA copy number (+5.8%, *p* = 0.047). *P. intermedia* increased from 3.393 to 3.665 log_10_ DNA copy number (+8.0%, *p* = 0.718), *C. rectus* remained nearly unchanged from 1.720 to 1.755 log_10_ DNA copy number (+2.0%, *p* = 0.942), *F. nucleatum* increased from 5.895 to 6.067 log_10_ DNA copy number (+2.9%, *p* = 0.143), and *S. mutans* increased from 2.098 to 2.306 log_10_ DNA copy number (+9.9%, *p* = 0.682).

## Discussion

This study evaluated the short-term effects of ODF on halitosis and oral pathogenic bacteria in comparison with the tongue cleaner. The results showed that H_2_ and CH3SH concentrations significantly decreased after ODF consumption (55.1% and 54.4%, respectively), and *S. mutans* levels also significantly decreased. In the tongue cleaner group, halitosis-related compounds concentrations showed a decreasing trend but were not statistically significant, while *T. forsythia* levels actually increased significantly.

The decrease in hydrogen concentration in the ODF group is considered to be due to the antimicrobial effect of the active ingredients in the film against hydrogen-producing bacteria in the oral and small intestinal cavity. In the etiological classification of halitosis, type 4 (blood-borne) can be identified using H_2_ (Aydin and Harvey-Woodworth, 2014; Aydin et al., 2016). Therefore, we included hydrogen as a halitosis indicator in this study. However, hydrogen is mainly produced through intestinal microbial fermentation and is a major indicator for diagnosing gastrointestinal diseases such as small intestinal bacterial overgrowth (SIBO) and carbohydrate malabsorption (Rana and Malik, 2014). A previous study has reported that SIBO and extra-oral halitosis show high correlation (Qian, 2024). In other words, hydrogen is not only a direct halitosis-causing substance, but it can be utilized as an indirect biomarker for evaluating extra-oral halitosis. One of the bacteria commonly found in SIBO is *Streptococcus* species, which appears in 71% of cases (Quigley and Abu-Shanab, 2010). In this study, we confirmed a significant reduction in *S. mutans* in the ODF group. According to the oral-gut axis theory, oral microorganisms can migrate to the gastrointestinal tract through saliva and influence intestinal microbial composition (Kunath et al., 2024). Therefore, the reduction of oral *Streptococcus* through ODF intake may have led to decreased colonization of the same bacterial genus in the small intestine, potentially resulting in reduced hydrogen production. However, to support this mechanism, future studies should include direct assessment of small intestinal microbiota and oral-gut microbial translocation pathways. According to recent studies, oral anaerobic bacteria, particularly *Klebsiella pneumoniae*, can produce significant amounts of H_2_ in the presence of glucose (Kanazuru et al., 2010). *S. acmella* extract, one of the active ingredients in the oral dissolving film, contains an essential oil called spilanthol. Previous studies have reported that spilanthol exhibits antimicrobial effects against *K. pneumoniae* similar to doxycycline (Arora et al., 2011). Therefore, ODF likely reduced H_2_ levels significantly by reducing anaerobic bacteria in the oral and small intestinal cavity. In contrast, the tongue cleaner group did not reach statistical significance despite a notable decrease of 44.41% (*p* = 0.264). This result is consistent with previous research demonstrating that physical tongue cleaning provides only transient effects, with VSC reduction lasting 15–100 min depending on the device used, and achieving only 33–42% reduction (Roldán et al., 2003). This demonstrates the temporary effect of physical removal and rapid bacterial recolonization.

H_2_S is produced by the degradation of sulfur-containing amino acids such as cysteine and methionine. H_2_S reduction in both groups did not reach statistical significance. Similarly, a previous study comparing a group that consumed tablets containing probiotics with a control group showed significant differences between groups in CH3SH but not in H_2_S (Lee et al., 2020). This suggests that H_2_S production involves more complex biochemical pathways compared to other gases, and there may be large inter-individual differences in basal sulfur-containing amino acid metabolism (Wu et al., 2022). The biosynthesis of methyl mercaptan is primarily produced by anaerobic bacteria in the oral cavity, but production by intestinal microorganisms has also been reported (Philipp et al., 2023; Harvey-Woodworth, 2013). The tongue cleaner group also showed a considerable decrease of 54.01% but remained at the statistical significance borderline (*p* = 0.079). The achievement of statistical significance only in the ODF group suggests that ODF affected not only the oral cavity but also the gastrointestinal tract.

No significant between-group differences were observed for halitosis parameters, which can be attributed to several factors. Both treatment modalities demonstrated meaningful within-group improvements. The ODF group showed significant reductions in H_2_ and CH3SH levels, while the tongue cleaner group also showed substantial reductions. The substantial individual variation was observed within each group. This made it difficult to detect between-group differences. The limited statistical power inherent in this pilot study design may have constrained our ability to identify subtle differences between treatments. Additionally, the study participants showed a relatively low distribution of baseline halitosis levels. This may have further limited the ability to detect dramatic between-group differences. Given these factors, the lack of between-group differences likely reflects the comparable therapeutic efficacy of both interventions rather than insufficient treatment effects. Future investigations should consider enrolling participants with higher baseline halitosis levels. This would enhance the discriminatory power of comparative analyses.

The clinical significance of these findings extends beyond statistical measures. In the ODF group, CH3SH levels decreased from 2.6 ng/10mL to 1.2 ng/10mL, representing a transition from clinically detectable oral malodor to slight perception levels (Kim and Kang, 2023). This improvement has important psychological implications, as patients with severe halitosis perception demonstrate significantly higher psychological distress scores compared to those with mild symptoms (Rani et al., 2022). ODF offers distinct advantages over traditional mechanical cleaning methods, including ease of use, portability, and the convenience of oral consumption without requiring additional equipment or technique training. However, potential limitations of ODF should be considered for clinical application. These may include patient compliance issues due to taste preference, possible gastrointestinal discomfort in sensitive individuals, and the need for regular consumption to maintain effects. Furthermore, the long-term safety profile of daily ODF consumption requires further investigation.

The significant decrease in *S. mutans* in the ODF group is believed to be due to the effects of probiotics, *S. acmella* extract, and xylitol. The *L. rhamnosus* strains included in the ODF consume oxygen and produce hydrogen peroxide, thereby changing the oxidation-reduction potential of the surrounding microenvironment (Jiang et al., 2016). They are also reported to increase the proportion of beneficial bacteria in the oral cavity and inhibit the growth of harmful bacteria (Park et al., 2023). Spilanthol contained in *S. acmella* extract exhibits excellent antibiofilm activity against *S. mutans* (Prachayasittikul et al., 2013). Additionally, xylitol has been reported to reduce *S. mutans* and prevent caries (Söderling, 2009). Furthermore, ODF consumption, unlike tongue cleaner use, is thought to have affected not only bacteria residing on the tongue but also bacteria residing on teeth. One previous study reported that consuming tablets containing kiwifruit powder was more effective in reducing total bacteria in tongue coating than tongue brushing (Matsumura et al., 2020). Therefore, ODF consumption caused specific activity inhibition and community changes against *S. mutans* in the oral cavity, significantly reducing its levels, suggesting the caries prevention potential of ODF.

Periodontal pathogenic bacteria belonging to the red complex (*P. gingivalis, T. forsythia, T. denticola*) and orange complex (*P. intermedia, C. rectus, F. nucleatum*) did not show significant decreases in the ODF group. This may be because the study period (7–10 days) was not long enough to observe significant changes in periodontal pathogenic bacteria. A previous study reported that when powder containing *Weissella cibaria* was fed to beagles for 2 weeks, four periodontal pathogenic species including *P. gingivalis* and *F. nucleatum* showed no significant changes and began to decrease only after 4 weeks (Do et al., 2019). Conversely, *T. forsythia* levels actually increased significantly in the tongue cleaner group, suggesting that mechanical cleaning of the tongue may lead to an increase in anaerobic bacteria that mainly reside subgingivally. Similarly, a study evaluating human oral microflora before and after 2 weeks of tongue scraper use reported that the average levels of anaerobic bacteria in saliva increased from 8.27 log10 cfu/mL to 8.34 log10 cfu/mL (Laleman et al., 2018).

Several limitations should be acknowledged. The study design limitations include the short duration (7–10 days), small sample size with insufficient statistical power, and inability to implement blinding due to the nature of interventions. Participant-related limitations encompass gender imbalance (no males in the control group) and relatively low baseline halitosis levels, which may limit generalizability. Future investigations should address these limitations through longer study periods, larger sample sizes with stratified randomization, and inclusion of intestinal microbiota analysis to better understand the oral-gut axis mechanisms observed in this study.

## Conclusion

This pilot study demonstrated that ODF use resulted in significant reductions in H_2_, CH3SH, and *S. mutans* levels, while tongue cleaner use showed trends toward improvement without achieving statistical significance. The significant T. forsythia increase in the tongue cleaner group suggests potential limitations of mechanical cleaning approaches. Although between-group differences were not statistically significant, ODF offers practical advantages as a convenient, dual-purpose intervention for halitosis management and caries prevention. These preliminary findings warrant validation through larger-scale, randomized controlled trials with extended follow-up periods to establish the long-term efficacy and safety profile of ODF therapy.

## Data Availability

The raw data supporting the conclusions of this article will be made available by the authors, without undue reservation.
